# Interactions of melatonin, reactive oxygen species, and nitric oxide during fruit ripening: an update and prospective view

**DOI:** 10.1093/jxb/erac128

**Published:** 2022-03-24

**Authors:** Francisco J Corpas, Marta Rodríguez-Ruiz, María A Muñoz-Vargas, Salvador González-Gordo, Russel J Reiter, José M Palma

**Affiliations:** Group of Antioxidants, Free Radicals and Nitric Oxide in Biotechnology, Food and Agriculture, Department of Biochemistry, Cell and Molecular Biology of Plants, Estación Experimental del Zaidín (Spanish National Research Council, CSIC), C/ Profesor Albareda, 1, 18008 Granada, Spain; Group of Antioxidants, Free Radicals and Nitric Oxide in Biotechnology, Food and Agriculture, Department of Biochemistry, Cell and Molecular Biology of Plants, Estación Experimental del Zaidín (Spanish National Research Council, CSIC), C/ Profesor Albareda, 1, 18008 Granada, Spain; Group of Antioxidants, Free Radicals and Nitric Oxide in Biotechnology, Food and Agriculture, Department of Biochemistry, Cell and Molecular Biology of Plants, Estación Experimental del Zaidín (Spanish National Research Council, CSIC), C/ Profesor Albareda, 1, 18008 Granada, Spain; Group of Antioxidants, Free Radicals and Nitric Oxide in Biotechnology, Food and Agriculture, Department of Biochemistry, Cell and Molecular Biology of Plants, Estación Experimental del Zaidín (Spanish National Research Council, CSIC), C/ Profesor Albareda, 1, 18008 Granada, Spain; Department of Cell Systems and Anatomy, Joe R. and Teresa Lozano Long School of Medicine, UT Health San Antonio, San Antonio, TX 78229, USA; Group of Antioxidants, Free Radicals and Nitric Oxide in Biotechnology, Food and Agriculture, Department of Biochemistry, Cell and Molecular Biology of Plants, Estación Experimental del Zaidín (Spanish National Research Council, CSIC), C/ Profesor Albareda, 1, 18008 Granada, Spain; University of Murcia, Spain

**Keywords:** Hydrogen peroxide, nitric oxide, nitrosomelatonin, melatonin, postharvest, ripening

## Abstract

Fruit ripening is a physiological process that involves a complex network of signaling molecules that act as switches to activate or deactivate certain metabolic pathways at different levels, not only by regulating gene and protein expression but also through post-translational modifications of the involved proteins. Ethylene is the distinctive molecule that regulates the ripening of fruits, which can be classified as climacteric or non-climacteric according to whether or not, respectively, they are dependent on this phytohormone. However, in recent years it has been found that other molecules with signaling potential also exert regulatory roles, not only individually but also as a result of interactions among them. These observations imply the existence of mutual and hierarchical regulations that sometimes make it difficult to identify the initial triggering event. Among these ‘new’ molecules, hydrogen peroxide, nitric oxide, and melatonin have been highlighted as prominent. This review provides a comprehensive outline of the relevance of these molecules in the fruit ripening process and the complex network of the known interactions among them.

## Teaser

Melatonin is an antioxidant with signaling properties that is especially relevant to fruit ripening. Melatonin crosstalk with NO and H_2_O_2_broadens the regulatory networks of these molecules.

## Introduction

Fruits are specialized organs whose function is to provide an appropriate environment for the formation and maturation of seeds that will be propagated by various procedures to preserve the species (Dardick and Callahan, 2014). Regardless of the different classifications of fruits, their ripening involves complex physiological processes that are associated with multiple changes at the genetic, proteomic, biochemical, and metabolic levels, which are highly coordinated (Klee and Giovannoni, 2011; Palma *et al.*, 2011; Karlova *et al.*, 2014; Lü *et al.*, 2018; Palma *et al.*, 2019; Aghdam *et al.*, 2020b). Fleshy fruits are a good example in which endogenous metabolic fluctuations can be translated into external changes that are easily observed at the phenotypic level, in many cases consisting of drastic changes in organoleptic features (e.g. color, emission of volatiles, and flavor) (Lalel *et al.*, 2003; Wu *et al.*, 2018).

Plants contain a wide variety of molecules that exert regulatory functions either independently or through their interactions with other molecules, acting as plant hormones. Among the classical phytohormones are auxins, cytokinins, gibberellins (GA), abscisic acid (ABA), and ethylene, but there are also other groups, including brassinosteroids (BR), salicylates, jasmonates, and strigolactones (Vanstraelen and Benková, 2012; Asgher *et al.*, 2017). Recently, different types of molecules previously considered toxic to cells have been found to exert signaling functions either directly or indirectly. Accordingly, molecules such as hydrogen peroxide (H_2_O_2_) and nitric oxide (NO), which are part of the metabolism of reactive oxygen species (ROS) and reactive nitrogen species (RNS), have also been shown to be regulators of plant cellular metabolism, participating in all stages of plant development including seed germination, root and plant development, stomatal movement, senescence, flowering, and fruit ripening, as well as in the mechanisms of response to adverse environmental conditions (Smirnoff and Arnaud, 2019; Liu *et al.*, 2020; Rodrigues and Shan, 2021; Corpas *et al.*, 2022; Gupta *et al.*, 2022). Other molecules could also be placed in the same category, such as hydrogen sulfide (H_2_S), which has recently been shown to exert regulatory functions in numerous processes, including fruit ripening (Gotor *et al.*, 2019; Corpas, 2019; Corpas *et al.*, 2021; Mishra *et al.*, 2021).

**Fig. 1.**
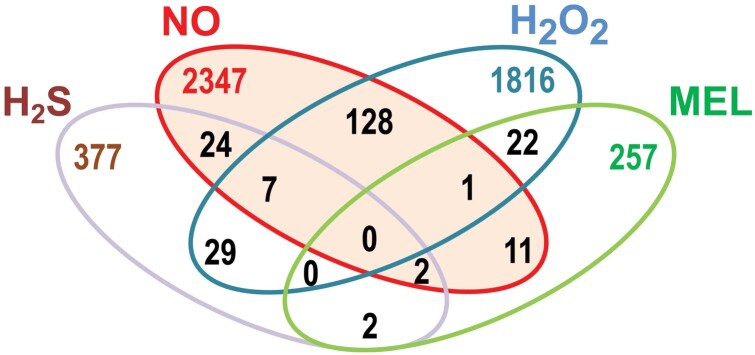
Venn diagram analysis of the number of publications on the different signal molecules, namely nitric oxide (NO), hydrogen peroxide (H_2_O_2_), hydrogen sulfide (H_2_S), and melatonin (MEL), related to fruits, found in the PubMed database in the period 1980–2022.

The hormone melatonin, which was discovered in higher plants in 1995 (Dubbels *et al.*, 1995; Hattori *et al.*, 1995) ,is a well-known regulatory molecule in mammals; in humans, for example, it influences numerous physiological and pathological processes such as circadian rhythms (Dominguez-Rodriguez *et al.*, 2010), metabolism (Korkmaz *et al.*, 2009), aging (Majidinia *et al.*, 2018), neurodegenerative diseases (Shukla *et al.*, 2019), and a wide range of cancers (González *et al.*, 2021; Moloudizargari *et al.*, 2021). Melatonin also has a broad spectrum of functions in higher plants (Zhao *et al.*, 2021; Arnao *et al.*, 2022; Hernández-Ruiz *et al.*, 2022). Consequently, melatonin has been defined as a ‘master regulator’ in animal and plant cells (Reiter *et al.*, 2010; Wang *et al.*, 2018; Arnao and Hernández-Ruiz, 2019, 2021a; C. Sun *et al.*, 2021), although the available information on melatonin in plants is still limited in comparison to that in animals. Figure 1 shows a Venn diagram analysis of the number of publications from the 1980s to date covering the different signaling molecules (NO, H_2_O_2_, H_2_S, and melatonin) as they relate to fruit physiology, with NO having the greatest number of publications, followed by H_2_O_2_, H_2_S, and melatonin. A small number of publications have simultaneously analyzed melatonin in combination with other signaling molecules, indicating that this is an emerging area that should be addressed.

The present review provides a framework of the relevance of melatonin in fruit ripening from the perspective of H_2_O_2_ and NO metabolism. Thus, melatonin, as a free radical scavenger, exerts regulatory actions over some ROS and RNS. The biochemical interactions among melatonin and these reactive species provide a promising new area of research due to the potential signaling functions of these interactions. Furthermore, the biotechnological significance of these molecules during fruit postharvest storage is discussed, as well as the effects of their exogenous application.

## Fleshy fruit ripening

Fleshy fruits are classically divided into two main categories according to their dependence on the ethylene profile and the respiratory burst: climacteric (e.g. apple, apricot, avocado, banana, melon, pear, persimmon, and tomato), which are dependent on ethylene and the respiratory burst, and non-climacteric (e.g. cherry, grape, orange, lemon, other citrus, olive, pepper, raspberry, and strawberry), which are not dependent on these factors (Cherian *et al.*, 2014; Chen *et al.*, 2018). However, fruit ripening also involves the integration of other molecules, including abscisic acid, auxin, jasmonic acid, and salicylic acid, which exert regulatory functions (Symons *et al.*, 2012; Kumar *et al.*, 2014; Hou *et al.*, 2018; Kou *et al.*, 2021; P. Li *et al.*, 2021; Alferez *et al.*, 2021). Recently, it was found that fruit ripening involves a physiological nitro-oxidative stress, which influences many subcellular processes such that some metabolic pathways are down-regulated whereas others are up-regulated (Chaki *et al.*, 2015; Rodríguez-Ruiz *et al.*, 2017; Corpas *et al.*, 2018a; Chu-Puga *et al.*, 2019; Palma *et al.*, 2019; González-Gordo *et al.*, 2019;  Zuccarelli *et al.*, 2021). Furthermore, accumulating data indicate that the exogenous application of some key molecules, such as NO, H_2_O_2_, or H_2_S, and most recently melatonin, has beneficial effects at different levels, including delay of fruit senescence, palliating chilling injury, ameliorating fungal decay, and improving nutritional quality. In many cases, all these molecules participate in complex signaling cascades that, in general, stimulate the enzymatic and non-enzymatic antioxidant systems.

## Melatonin

Melatonin (*N*-acetyl-5-methoxytryptamine) is generated from the amino acid tryptophan. In vertebrates, melatonin is the main secretory product of the pineal gland located in the brain and is probably best known for its influence on sleep. However, melatonin has multiple regulatory functions in physiological and pathological conditions (Shukla *et al.*, 2019; Ma *et al.*, 2020; Back, 2021). In plant cells, this molecule is referred to as phytomelatonin and it has phytohormonal actions in higher plants, which include its antioxidant properties. A putative melatonin receptor in the plasma membrane designated CAND2/PMTR1 (Candidate G-protein coupled receptor 2/Phytomelatonin receptor 1), which participates in the signaling mechanisms related to stomatal closure of *Arabidopsis thaliana*, has been identified. This signaling involves a cascade of signals, including H_2_O_2_, Ca^2+^ influx, and K^+^ efflux, in stomatal guard cells (Wei *et al.*, 2018). More recently, new data obtained using confocal microscopy and Cand2-defective Arabidopsis mutants indicate that the Cand2 protein is actually localized in the cytosol and may not be a G protein that mediates melatonin-induced defense (Back and Lee, 2020). Therefore, the presence of a melatonin receptor on the plasma membrane in higher plant cells obviously remains an open question. It is, however, well recognized in higher plants that melatonin participates in regulatory functions at different levels, such as promoting lateral root growth, delaying senescence, flowering, and fruit ripening, ameliorating iron deficiency, and mediating the response to environmental stresses (Korkmaz *et al.*, 2014; Zhang *et al.*, 2015; Zhu *et al.*, 2019; Zhao *et al.*, 2019; Arnao and Hernández-Ruiz, 2020; Siddiqui *et al.*, 2020), and that these regulatory processes involve molecules such as H_2_O_2_, NO, or H_2_S that have signaling properties, although the existence of a receptor is still under analysis (S. Li *et al.*, 2021b; Pardo-Hernández *et al.*, 2021; Singh *et al.*, 2022).

## Reactive oxygen species: H_2_O_2_ as a signal molecule

ROS are a family of molecules generated during the reduction of molecular oxygen. They include hydrogen peroxide (H_2_O_2_), superoxide anion (O_2_^•–^), hydroxyl radical (^•^OH), and other species that do not involve electron gains, such as singlet oxygen (^1^O_2_). In higher plants, the main sources of ROS are the electron transport chains of chloroplasts and mitochondria, as well as peroxisomes, which are a particularly important source of H_2_O_2_ due to the β-oxidation and photorespiration pathways (Corpas *et al.*, 2020). Additionally, there are other minor cellular sites of ROS production including the cytosol, plasma membrane, and cell wall (Corpas *et al.*, 2015; Podgórska *et al.*, 2017; Kámán-Tóth *et al.*, 2019). Furthermore, the uncontrolled overproduction of ROS, such as occurs as a consequence of adverse environmental conditions, can trigger oxidative damage to the various cellular macromolecules, causing their dysfunction (Møller *et al.*, 2007). However, some ROS have signaling properties, in particular H_2_O_2,_ which has been extensively studied (Exposito-Rodriguez *et al.*, 2017; Foyer, 2018, 2020; Smirnoff and Arnaud, 2019; Nazir *et al.*, 2020; Liu *et al.*, 2021; Zhang *et al.*, 2021; Zentgraf *et al.*, 2022). Recent reports have identified two H_2_O_2_ plasma membrane receptors, designated leucine-rich repeat (LRR) receptor protein kinase HPCA1 (Wu *et al.*, 2020) and LRR-receptor-like kinase (RLK) protein HSL3 (Liu *et al.*, 2020), which sense the apoplastic content of H_2_O_2_ and initiate a cascade of signals as a response mechanism to different exogenous stimuli (Foyer *et al.*, 2020; Singh *et al.*, 2022). Likewise, during the plant immune response, O_2_^•–^ generation is controlled by a receptor-like cytoplasmic kinase (RLCK)-mediated phosphorylation of respiratory burst oxidase homolog D (RBOHD) (P. Li *et al*, 2021a; Singh *et al.*, 2022).

## Reactive nitrogen species: nitric oxide as a signal molecule

The discovery that plant cells have the capacity to generate the free radical nitric oxide (^•^NO) opened a new area of research (Kolbert *et al.*, 2019). Unlike animals, in which the enzymatic NO source from the amino acid l-arginine involves a group of well-characterized enzymes named nitric oxide synthases (NOSs), in higher plants, the enzymatic source remains undefined, although it is generally accepted that there are two main routes: (i) a reductive pathway from nitrate and nitrite that is mediated by nitrate reductase (NR), and (ii) an oxidative pathway from l-arginine through a NOS-like activity, designated thus because it has the same biochemical requirements as animal NOS (Astier *et al.*, 2018; Corpas *et al.*, 2022). In higher plants, the main NO sources are the cytosol, peroxisomes, chloroplasts, and mitochondria.

Metabolism of NO leads to the formation of derived molecules, called RNS, which include nitrogen dioxide (^•^NO_2_), peroxynitrite (ONOO^–^), and *S*-nitrosoglutathione (GSNO), among others (Corpas, 2017). ONOO^–^ is a highly reactive molecule and also a strong oxidizing and nitrating agent (Ferrer-Sueta *et al.*, 2018) that is formed by the chemical reaction between two radicals, ^•^NO and O_2_^•–^, with a very high rate constant (~10^10^ M^–1^ s^–1^), even higher than the rate constant for O_2_^•–^ dismutation by the CuZn superoxide dismutase (SOD) enzyme, which is 2 × 10^9^ M^–1^ s^–1^ (Gray and Carmichael, 1992). This characteristic guarantees that when both radicals are simultaneously present in any plant subcellular location, ONOO^–^ will be generated. On the other hand, GSNO is also generated by the binding of NO to the thiol group of the reduced form of glutathione (GSH, γ-l-glutamyl-l-cysteinylglycine), which is considered to be the main *S*-nitrosothiol in plant cells and whose content is regulated by the enzyme GSNO reductase (Lee *et al.*, 2008; Corpas *et al.*, 2013). These two examples, ONOO^–^ and GSNO, demonstrate the close relationship between ROS and RNS metabolism.

In plants, RNS regulate protein functions by post-translational modifications (PTMs). Tyrosine nitration is an irreversible process that usually causes inhibition of the target proteins (Radi, 2013; Mata-Pérez *et al.*, 2016), whereas *S*-nitrosation in the thiol group of key cysteine residues, which is reversible, can either up-regulate or down-regulate enzyme functions (Corpas *et al.*, 2008; Gupta *et al.*, 2020). To date, it has been demonstrated that the main antioxidant enzymes in plant cells, such as catalase, ascorbate peroxidase (APX), monodehydroascorbate reductase, and SODs, can undergo either nitration, *S*-nitrosation, or both (Begara-Morales *et al.*, 2014a, 2015, 2016;  Palma *et al.*, 2020). Under adverse environmental conditions, RNS are also overproduced in an uncontrolled way and trigger dysfunction in macromolecules, causing nitrosative stress; ONOO^–^ has a particular relevance for protein nitration (Corpas and Barroso, 2014). In a mirrored manner to H_2_O_2,_ which is the most studied ROS participating in signaling functions in plant cells, NO is the most highly investigated RNS (Corpas *et al.*, 2018b; Kohli *et al.*, 2019; Gupta *et al.*, 2022).

## Chemical and biochemical interactions of melatonin with ROS and RNS

**Fig. 2.**
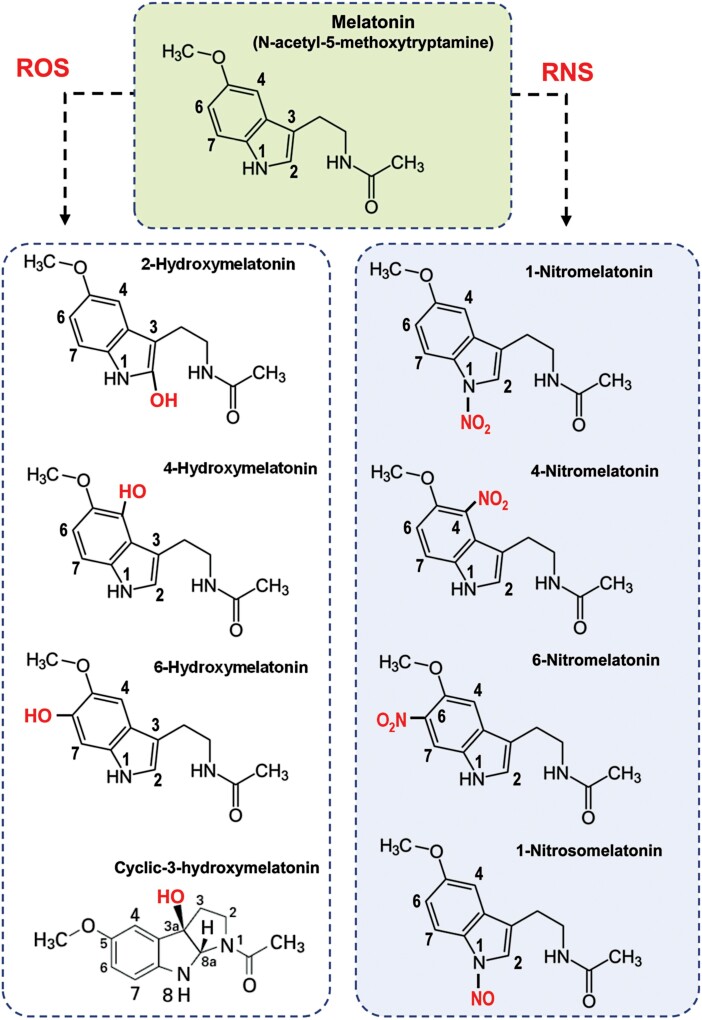
Melatonin-derived metabolites resulting from the interaction of melatonin with ROS and RNS. The reactions involve the addition of a hydroxyl group (-OH) in position 2, 4, or 6; the addition of NO (1-nitrosomelatonin); or the addition of a nitro group (NO_2_) in position 1, 4, or 6.

It is well recognized that melatonin is a potent free radical scavenger (Reiter *et al.*, 2001; Sofic *et al.* 2005; Tan *et al.*, 2007; Galano *et al.*, 2013; Zhang and Zhang, 2014) that can diffuse through biological membranes, exerting its antioxidant capacity in the different subcellular compartments. Melatonin reacts with different ROS or RNS to form a family of molecules that involve either the addition of a hydroxyl group (-OH) in position 1, 4, or 6 to generate hydroxymelatonin; the addition of a nitro group (-NO_2_) in position 1, 4, or 6 to generate *N*-nitromelatonin (Kirsch and de Groot, 2009); or the addition of NO in position 1 to generate 1-nitrosomelatonin (NOMel) (Blanchard *et al.*, 2000) (Fig. 2). Among the different hydroxymelatonin metabolites, 4-hydroxymelatonin (4-OHM) is an excellent peroxyl radical (ROO^•^) scavenger, whereas 2-hydroxymelatonin (2-OHM) is predicted to have low antioxidant protection. Under *in vitro* conditions at physiological pH, it was shown that 4-OHM reacted with ROO^•^ ~200 times faster than trolox (an analog of vitamin E used to measure antioxidant capacity). Furthermore, 4-OHM was predicted to have a higher antioxidant capacity than natural antioxidants present in different fruits, such as gallic and ellagic acids in blueberry, blackberry, strawberry, plum, or grape (Pérez-González *et al.*, 2017). In humans, mice, and rats, another metabolite of melatonin has been described, namely cyclic 3-hydroxymelatonin, which was found to have a higher hydroxyl radical (^•^OH) scavenging capacity than either melatonin or vitamin C (Tan *et al.*, 2014). To corroborate the formation of these compounds, an *in vitro* assay using 5 mM melatonin in the presence of 5 mM 3-morpholinosydnonimine (SIN-1, a peroxynitrite donor that simultaneously generates equimolar amounts of O_2_^•–^ and ^•^NO) was carried out at 37 °C for 60 min in the dark. Subsequently, the profile of compounds was analyzed by liquid chromatography-tandem mass spectrometry (LC-MS/MS), and all the hydroxy-, nitro-, and nitrosomelatonin derivatives were found.

Byeon *et al.* (2015) performed a systematic analysis using a LC-MS/MS approach to evaluate the content of melatonin and some of its hydroxy-derived molecules in 24 plant species. This study found that in most plant species the melatonin concentration is ~1 ng g^–1^ fresh weight (FW). In contrast, the content of 2-OHM is ~6 ng g^–1^ FW. A deeper analysis of the hydroxyl forms of melatonin in the selected plants indicated that the predominant form was 2-OHM (99%), followed by 4-OHM (~0.5%), with 6-hydroxymelatonin being undetected. Unfortunately, any melatonin molecule related to NO was not analyzed in this study.

Melatonin is known to react with peroxynitrite, and it could be expected that in a cellular environment, this would be a mechanism of protection against protein nitration processes that are usually associated with a down-regulation in the function of the affected protein (Begara-Morales *et al.*, 2013; Radi, 2013; Ferrer-Sueta *et al.*, 2018; Muñoz-Vargas *et al.*, 2018; Corpas *et al.*, 2021). It is important to consider that the formation of peroxynitrite is usually associated with the overproduction of both ^•^NO and O_2_^•–^, whose coupling activity is very high; as a result, the product, peroxynitrite, has major negative effects where it is generated. Thus, the interaction of peroxynitrite with melatonin is an additional mechanism of protection of proteins against nitration. particularly under stress conditions; this deserves to be further investigated.

## Interaction among *N*-nitrosomelatonin, NO, and *S*-nitrosothiols

**Fig. 3.**
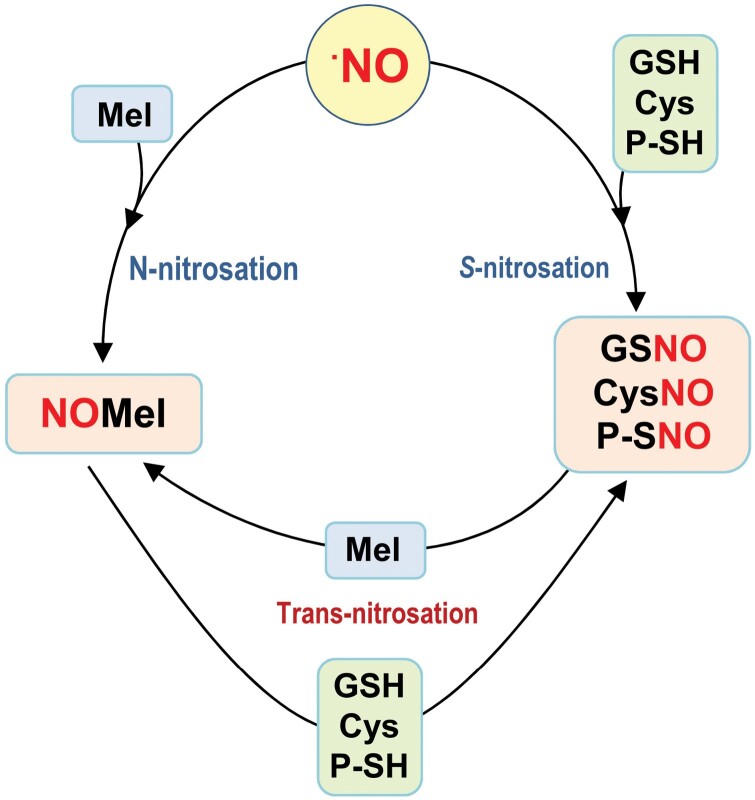
Simple model of melatonin (Mel) nitrosation, *S*-nitrosation of glutathione (GSH), cysteine (Cys), or protein thiol (P-SH), and *trans*-nitrosation. Nitric oxide (NO) interacts with Mel, GSH, Cys, and P-SH to generate nitrosomelatonin (NOMel), *S*-nitrosoglutathione (GSNO), *S*-nitrosocysteine (CysNO), or nitrosated protein (P-SNO), respectively, which can undergo *trans*-nitrosation processes.

Early *in vitro* studies evaluated the capacity of NOMel to release NO. Subsequently, these assays were completed under physiological conditions where NOMel, in the presence of reducing compounds such as ascorbate, released NO and melatonin (De Biase *et al.*, 2005). The physiological relevance of this process is similar to that exerted by *S*-nitrosothiols of low and high molecular weight, including GSNO, nitrosocysteine, and *S*-nitrosated proteins, which are also NO-releasing compounds. For example, in the presence of reductants (ascorbate and GSH, and Cu^2+^), GSNO decomposes to produce ^•^NO and oxidized glutathione (Gorren *et al.* 1996; Noble *et al.*, 1999; Holmes and Williams 2000; Smith and Dasgupta 2000). In 30-day-old Arabidopsis plants, exogenous GSNO applied to the root system was available to release NO and modulate up to 1945 genes that were expressed differently in leaves and roots, with 114 genes being exclusive to one of these organs, indicating the capacity of the GSNO to move long distances through the vascular system (Begara-Morales *et al.*, 2014b). Based on this property, Fig. 3 shows a working model in which NOMel could release NO and mediate a process of *S*-nitrosation of GSH, free cysteine, and thiol groups of proteins. At the same time, these *S*-nitrosothiols release NO and, by a ­*trans*-nitrosation process, mediate the formation of NOMel (Peyrot *et al.*, 2006; Berchner-Pfannschmidt *et al.*, 2008; Hickok *et al.*, 2012; Mukherjee, 2019; Hardeland, 2021). These mechanisms could be considered a cellular strategy to extend the functional actions of the involved molecules in the different subcellular compartments in which they are generated. Consequently, a close relationship among all these molecules with the capacity to carry and release NO should be anticipated, with this being a long-distance signaling mechanism (Singh *et al.*, 2016).

In higher plants, the information about the formation of hydroxy- and nitromelatonin metabolites is, to the best of our knowledge, limited, and it is mostly based on *in vitro* and *in vivo* studies of animal cells (Hardeland, 2021). However, it could be expected that these compounds should have analogous functions in plant cells. In addition to the direct interactions between melatonin and the different ROS and RNS, there are also some mechanisms in plants that mediate the ­conversion of melatonin into 2-OHM and cyclic 3-hydroxymelatonin through the enzymatic action of melatonin 2-hydroxylase (M2H) and melatonin 3-hydroxylase (M3H), respectively (Lee and Back, 2016; Lee *et al.*, 2016). Thus, some genetic studies in rice plants using RNA interference approaches to down-regulate the expression of M2H caused an increase the content of melatonin, conferring a higher tolerance to diverse stresses including cadmium, senescence, salt, and tunicamycin (Choi and Back, 2019). Similarly, treatment with 2-OHM induced plant defense genes in Arabidopsis, although to a smaller extent than melatonin (Byeon *et al.*, 2015), and in rice (*Oryza sativa*) 2-OHM triggered resistance against cold and drought stress (Lee and Back, 2016, 2019).

In cassava (*Manihot esculenta*) plants, there are obvious protein interactions among cytosolic ascorbate peroxidase (MeAPX2) and two cytosolic isozymes of the melatonin biosynthesis, tryptophan decarboxylase (MeTDC2) and *N*-aceylserotonin *O*-methyltransferase (MeASMT2), which provide a higher antioxidant capacity against H_2_O_2_ (Bai *et al.*, 2020). This observation prompts several questions focused on the molecular mechanism underlying how these protein interactions (MeAPX2–MeTDC2 and MeAPX2–MeASMT2) occur, where they take place, and whether they increase the APX activity and therefore provide greater protection against high concentrations of H_2_O_2_.

In higher plants, some experimental studies have reported an interaction between melatonin and NO (Y. Sun *et al.*, 2021). In sunflower (*Helianthus annuus* L.) seedlings subjected to salinity stress, the exogenous application of 15 µM melatonin altered the content of NO, O_2_^•–^, and ONOO^–^, and consequently the modulation of CuZn-SOD and Mn-SOD as well as protein tyrosine nitration (Arora and Bhatla, 2017). Recently, using 3-day-old Arabidopsis seedlings as a model, Singh *et al.* (2021) estimated the NO release capacity of two compounds, 250 μM GSNO and NOMel, applied through the root system, and evaluated the NO content in green cotyledons by confocal laser scanning microscopy. The results showed that NOMel is more efficient than GSNO in releasing NO. Consequently, these data indicate that both NOMel and GSNO have the capacity to travel through the vascular system and release NO in other parts of the plant, as was previously proposed (Airaki *et al.*, 2011; Begara-Morales *et al.*, 2014b; Singh *et al.*, 2016).

## Crosstalk among melatonin, H_2_O_2_, and NO in fruit ripening and postharvest storage

Knowledge of the mechanism of regulation among melatonin, NO and H_2_O_2_ during fruit ripening is in a nascent phase, especially due to the fact that there are earlier unresolved issues such as the identity of the genes involved in the melatonin biosynthesis pathway, as well as how the NO is generated. Since the first descriptions of the presence of melatonin in plants (Dubbels *et al.*,1995; Hattori *et al.*, 1995), interest in this molecule in the field of plant physiology has grown exponentially. This is especially due to its antioxidant properties, as well as its regulatory functions affecting both gene and protein expression, enzyme activities, and their crosstalk with different phytohormones (Arnao and Hernández-Ruiz, 2021b; Arnao *et al.*, 2022). Likewise, the study of the interactions of endogenous melatonin with ROS and RNS in higher plants has also been increasing, mainly based on the biochemical information established from animal studies that have provided basic knowledge in this field. However, research studies of the interactions between melatonin with both ROS and RNS during fruit ripening are still scarce (Aghdam *et al.*, 2022), possibly due to the low levels of endogenous melatonin in fruits, which make it difficult to identify the related melatonin metabolites. For example, a comparative analysis of the melatonin content of the most consumed horticultural fruits worldwide, pepper (*Capsicum annuum* L.) and tomato (*Solanum lycopersicum* L.), which are representative examples of non-climacteric and climacteric fruits, respectively, indicated that the melatonin concentration in red pepper fruits of six cultivars ranged from 5 ng g^–1^ to 12 ng g^–1^ FW, whereas in red tomato fruits of seven cultivars, the melatonin concentration ranged from 0.6 ng g^–1^ to 15 ng g^–1^ FW (Riga *et al.*, 2014). A similar situation is apparent concerning studies on the metabolism of endogenous NO in fruits, about which information is also very limited (Corpas *et al.*, 2018a), although in the case of ROS metabolism there is more information available.

Other challenges in delving into the regulatory mechanisms at the genetic level are to identify all the genes/enzymes involved in melatonin and NO biosynthesis. In the case of melatonin, its synthesis from the amino acid tryptophan in higher plants seems to involve six enzymes that are present in different subcellular compartments, namely tryptophan decarboxylase (TDC), tryptamine 5-hydroxylase (T5H), tryptophan hydroxylase (TPH), serotonin *N*-acetyltransferase (SNAT), *N*-acetylserotonin methyltransferase (ASMT), and caffeic acid *O*-methyltransferase (COMT). However, not all genes/enzymes have been identified in all plants (Aghdam *et al.*, 2022 and references therein) suggesting the existence of diverse biosynthesis pathways. In the case of NO, its biosynthetic pathway is even more disputed (Corpas *et al.*, 2022 and references therein). It would be of great interest if any of the enzymes involved in melatonin biosynthesis were found to be targets of NO-derived PTMs such as *S*-nitrosation and nitration, although, to our knowledge, this information has not yet been uncovered.

Consequently, the majority of the studies on fruits have been carried out after the exogenous application of either melatonin, NO, or H_2_O_2_. The few results reported indicate that these molecules regulate the ripening process, either slowing or accelerating it, or provide beneficial effects during postharvest storage; as a result, these compounds could be used as tools for biotechnological approaches to maintain the quality of the fruits as well as protecting them against infections by pathogens or chilling damage associated with postharvest storage. It should be pointed out, however, that the effects of these molecules on fruit ripening depend on the type of fruit (climacteric or non-climacteric) and the dose and duration of the treatment, among other parameters that must be optimized. Table 1 summarizes representative examples of climacteric and non-climacteric fruits treated with melatonin, H_2_O_2_, or NO and the beneficial effects of these treatments, such as extending postharvest storage life or preserving nutritional quality. However, it seems evident that the metabolic triangle constituted by melatonin, NO, and H_2_O_2_ has a common characteristic that implies the activation of both enzymatic and non-enzymatic antioxidant systems (Tan *et al.*, 2015; Chumyam *et al.*, 2019; Zuccarelli *et al.*, 2021). This may serve to control the overproduction of ROS and RNS that could trigger uncontrolled nitro-oxidative stress resulting in an alteration in the quality of the fruits, in terms of both their external appearance and their organoleptic qualities (aroma, flavor, acidity, sweetness, etc.).

**Table 1.** Representative examples of the beneficial effects triggered by exogenous molecules with signaling properties (melatonin, H_2_O_2_, and NO) in fruits to extend postharvest life or to preserve nutritional quality

Fruit	Concentration	Main effects	Reference
**Melatonin**			
Peach (*Prunus persica* L.)	0.1 mM	Delays postharvest senescence by lowering O_2_^•–^ and H_2_O_2_ accumulation. Higher AA accumulation and increased activity of catalase, SOD, and APX	Gao *et al.*, (2016)
Grapevine (*Vitis vinifera* × *labrusca*).	0.2 mM	Stimulates ripening by increasing the levels of ABA, H_2_O_2_, and ethylene	Xu *et al.*, (2018)
Pear (*Pyrus communis* L.)	0.1 mM	Delays postharvest senescence and induces NO accumulation. Higher *NOS-like* gene expression and enzyme activity. Lower *ACS*, *ACO*, *PG*, and *Cel* genes expression	Liu *et al.*, (2019)
Pear (*Pyrus communis* L.)	0.1 mM	Induces anthocyanin accumulation through the H_2_O_2_ generated by RBOHF	H. Sun *et al.*, (2021)
Sweet cherry (*Prunus avium* L.)	0.1 mM	Higher endogenous melatonin accumulation. Higher SOD, CAT, APX, and GR enzyme activity. Higher ascorbate and GSH accumulation. Higher membrane integrity. Lower electrolyte leakage and MDA accumulation. Lower O_2_^•–^ and H_2_O_2_ accumulation	Wang *et al.*, (2019)
Sweet cherry (*Prunus avium* L. var Prime Giant)	0.01 and 0.1 mM	Delays ripening by modulating the contents of endogenous hormones, mainly ABA and auxin	Tijero *et al.*, (2019)
Sweet cherry (*Prunus avium* L.)	0.50 and 0.1 mM	Treatment of leaves treated with melatonin improved the antioxidant content of sweet cherry fruit	Xia *et al.*, (2020)
Jujube (*Ziziphus jujuba* Mill.)	25 µM	Higher APX and GR enzyme activity. Higher ascorbate and GSH accumulation. Lower PG and PME enzymes activity, maintaining firmness	Tang *et al.*, (2020)
Pomegranate (*Punica granatum* L.)	0.1 mM	Higher NADPH accumulation. Higher APX, GR, G6PDH, 6PGDH, and PAL enzyme activity. Higher AOX gene expression. Higher phenol and anthocyanin accumulation and DPPH-scavenging capacity. Higher AA and GSH accumulation. Lower AAO enzyme activity.	Aghdam *et al.*, (2020a)
Mango (*Mangifera indica* L.)	0.2 mM	Delays the ripening process. Decreases the contents of H_2_O_2_ and MDA in the exocarp of the fruit	Dong *et al.*, (2021)
Apple (*Malus domestica* L. Borkh)	1 mM	Reduces ethylene production. Increase the activity of catalase, SOD and peroxidase and keeps apple quality during postharvest storage	Onik *et al.*, (2021)
Blueberry (*Vaccinium corymbosum* L.)	1 mM	Reduces qualitative decay and improves antioxidant system (catalase, SOD, APX, ascorbate, polyphenols, anthocyanins, and flavonoids) during cold storage	Magri and Petriccione, (2022)
Kiwifruit (*Actinidia chinensis*)	0.1 mM	Palliates chilling injury during cold postharvest storage by inhibition of lignin metabolism and increasing the activity of antioxidant enzymes and the content of soluble antioxidants (ascorbate and GSH)	Jiao *et al.*, (2022)
Tomato (*Solanum lycopersicum*)	0.5 mM	Promotes ripening of postharvest fruit through DNA methylation of ethylene-signalling genes	Shan *et al.*, (2022)
**H**_**2**_ **O**_**2**_			
Melon (*Cucumis melo* L.)	20 mM	Treatment of melon plants increases the soluble sugar content in leaves and fruits, thus improving the fruit quality. Increases photosynthetic activity and the activities of chloroplastic and cytosolic fructose-1,6-bisphosphatase, sucrose phosphate synthase, and invertases	Ozaki *et al.*, (2009)
Longan (*Dimocarpus longan* Lour)	1.96 mM	Increases the activities of pulp PLD, lipase, and LOX. Destroys longan pulp membrane structure and increases cell membrane permeability	Lin *et al.*, (2019)
Guava (*Psidium guajava* L.)	250 mM	Reduces enzymatic browning of freshly cut fruit by reducing PPO and POD activities. Stimulates the peroxiredoxin/thioredoxin system	Chumyam *et al.*, (2019)
Kyoho grape *(Vitis vinifera × Vitis labrusca)*	300 mM	Promotes early ripening. Affects the gene expression of *HSP*, *GDSL*, *XTH*, and *CAB1*, involved in oxidative stress, cell wall deacetylation, cell wall degradation, and photosynthesis, respectively.	Guo *et al.*, (2020)
Mango (*Mangifera indica* L.)	20 mM	Treated mango plants have fruits with a higher content of total sugar, phenol, and carotenoids	Mostafa, (2021)
Tomato (*Solanum lycopersicum* L. cv. Verty F_1_)	100 mM	Increases tomato fruit firmness, decreases water-soluble pectin and expression of cell-wall-related genes, polygalacturonase, and pectate lyase. Maintains morphological and biochemical quality of tomato fruits during postharvest storage	Torun and Uluisik, (2022)
**NO**			
Strawberry (*Fragaria × ananassa* Duch.)	5 µM sodium nitroprusside solution	Extends postharvest life	Wills *et al.*, (2007); Zhu and Zhou, (2007)
Peach fruit (*Prunus persica* L. cv. Xiahui 6)	10 ppm NO gas	Delays the ripening process. Affects sucrose metabolism by changing the expression of related genes	Han *et al.*, (2018)
Jujube (*Ziziphus jujuba* Mill.)	20 ppm NO gas	Retards cell wall degradation	Zhao *et al.*, (2019)
Sweet pepper (*Capsicum annuum* L. cv. Melchor)	5 ppm NO gas	Delays fruit ripening. Increases ascorbate content, protein nitration, and *S*-nitrosation. Decreases catalase and APX activities	Rodríguez-Ruiz *et al.*, (2017); González-Gordo *et al.*, (2019)
Tomato (*Solanum lycopersicum* L. cv. ‘Micro-Tom’)	300 ppm NO gas	Promotes ascorbate biosynthesis and intensifies protein *S*-nitrosation and nitration. Affects carotenoid, tocopherol, and flavonoid metabolism	Zuccarelli *et al.*, (2021)
Melon (*Cucumis melo* L.)	100 ppm NO gas	Enhances postharvest disease resistance to the fungus *Alternaria alternata* by postponing ethylene biosynthesis	Wei *et al.*, (2021)

AA, ascorbic acid; AAO, ascorbic acid oxidase; AOX, alternative oxidase; ABA, abscisic acid; ACS, 1-aminocyclopropane-1-carboxylic acid (ACC) synthase; ACO, ACC oxidase; APX, ascorbate peroxidase; CAB1, chlorophyll *a*-*b* binding protein; CAT, catalase; Cel, cellulose; DPPH, 2,2-diphenyl-1-picrylhydrazyl; GDSL, GDSL-motif esterase/lipase; G6PDH, glucose-6-phosphate dehydrogenase; GR, glutathione reductase; GSH, reduced glutathione; HSP, heat shock protein; LOX, lipoxygenase; MDA, malondialdehyde; NOS, NO synthase; PG, polygalacturonase; 6PGDH, 6-phosphogluconate dehydrogenase; PLD, phospholipase D; POD, peroxidase; PPO, polyphenol oxidase; RBOHF, respiratory burst oxidase homolog F; SOD, superoxide dismutase; XTH, xyloglucan endotransglucosylase/hydrolase.

In pepper fruits, treatment with NO gas causes delayed ripening, which is accompanied by a modulation of the ROS metabolism characterized by an elevation in ascorbate content as a consequence of an increase in the expression and activity of the last enzyme of its biosynthesis pathway, the mitochondrial enzyme l-galactono-1,4-lactone dehydrogenase (GalLDH) (Rodriguez-Ruiz *et al.*, 2017). Likewise, the NO-treated fruits had a higher GSH content, higher APX and lipoxygenase activities, lower lipid peroxidation, and lower O_2_^•–^-generating NADPH oxidase activity (González-Gordo *et al.*, 2019, 2020). Interestingly, a higher content of nitrated proteins was apparent, particularly the peroxisomal enzyme catalase, whose activity decreased (Chaki *et al.*, 2015). These observations related to APX and catalase activity are in good agreement with the previously reported effect of NO-derived PTMs, *S*-nitrosation, and nitration on these enzymes in other plant species (Begara-Morales *et al.*, 2014a; Palma *et al.* 2020). Similarly, the exogenous application of NO to tomato at the pre-climacteric stage suppressed the activity of antioxidant enzymes, increased protein *S*-nitrosation and nitration, and favored the accumulation of ascorbate and flavonoids (Zuccarelli *et al.*, 2021). Recently, it has been shown that melatonin exerts an epigenetic regulation through DNA methylation of ethylene signaling genes, which promotes the ripening of tomato fruit during postharvest storage (Shan *et al.*, 2022). This observation suggests a scenario to be addressed in future investigations.

The cascade of events that takes place when any of these molecules is applied exogenously has been the subject of many studies because there are other elements involved, such as the type of fruit, the involvement of phytohormones such as ethylene, or the state of preservation of the fruit, for example, at low temperature. For example, in pear fruits, the exogenous application of melatonin inhibits the synthesis of ethylene, which seems to be mediated by NO (Liu *et al.*, 2019), since this molecule can inhibit key enzymes in the ethylene biosynthesis pathway, such as *S*-adenosyl methionine synthetase, 1-aminocyclopropane-1-carboxylic acid (ACC) synthase, and ACC oxidase (Palma *et al.*, 2019). In the case of non-climacteric fruits, the ripening process is essentially modulated by ABA, which mediates the accumulation of anthocyanins and sugars. For example, in sweet cherry, exogenous melatonin delays fruit ripening, counteracting the effect of ABA, since it affects the balance of other involved phytohormones such as cytokinins, jasmonic acid, and salicylic acid (Tijero *et al.*, 2019; Michailidis *et al.*, 2021).

## Conclusions and future perspectives

As in mammals, melatonin is a multifunctional molecule in higher plants and specifically in fruits, where it exerts numerous beneficial functions as a protectant against biotic and abiotic stresses when it is exogenously applied. Melatonin has antioxidant properties, since it reacts with both ROS and RNS, although the information available on the derived molecules is scarce in higher plants and even non-existent in relation to the ripening of fruits, a process that is characterized by an important nitro-oxidative metabolism.

**Fig. 4.**
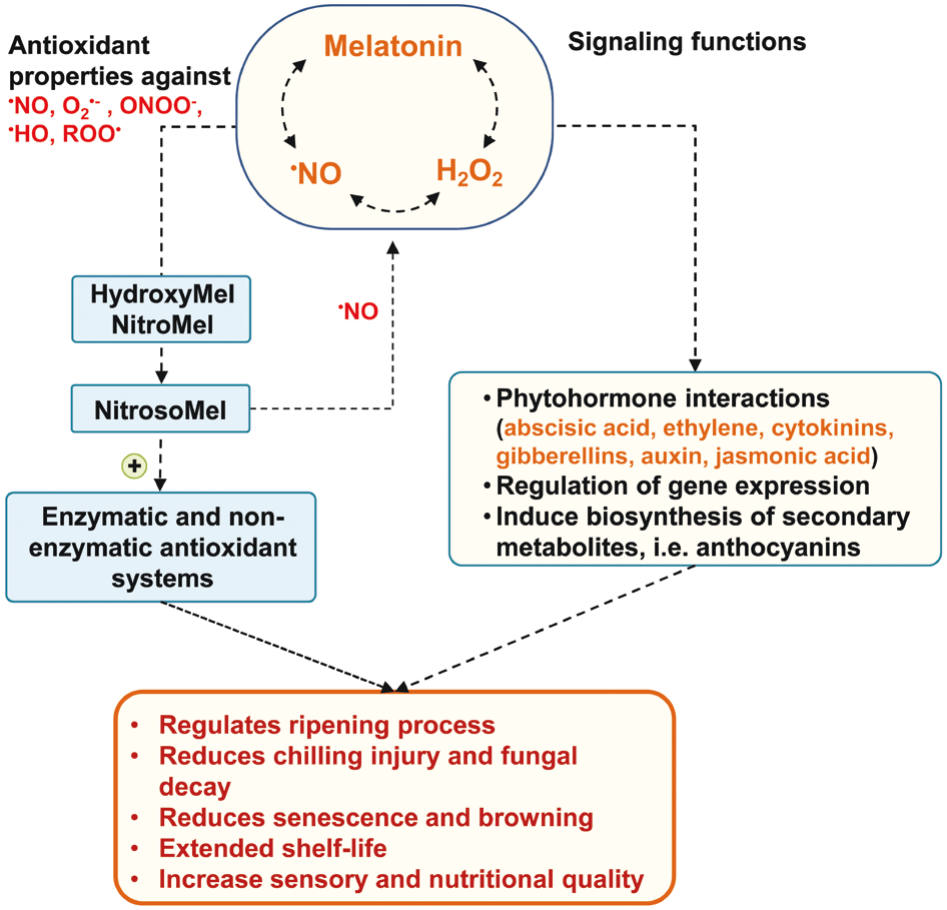
Overview of the cascade of signals triggered by the application of exogenous melatonin (Mel), NO, or H_2_O_2_ to modulate fruit ripening and quality.

Future research should focus on the interactions and functions of these molecules, although a major technical challenge is their identification and specific localization, considering that they are endogenously generated at very low concentrations. Unquestionably, the exogenous application of melatonin has been shown to be a powerful biotechnological tool, since it exerts beneficial effects either directly as an antioxidant molecule, or by acting as a signaling molecule that acts upstream of H_2_O_2_ and NO. Furthermore, the interaction of melatonin with NO to generate nitrosomelatonin, a molecule that can release NO in the presence of reductants such as ascorbate, opens new research lines related to the complex crosstalk between these molecules. On the other hand, the use of exogenous melatonin to provide beneficial effects during postharvest storage could be considered as a novel biotechnological tool for application in the horticultural industry. However, it is important to note that the melatonin concentration, the time of exposure, and the means of application (by immersion, spraying, or other methods) should be optimized for each type of fruit. Figure 4 illustrates the cascade of signals mediated by the crosstalk among melatonin, NO, and H_2_O_2_ during fruit ripening, which, in general, stimulates antioxidant capacity through the activation of enzymatic and non-enzymatic systems as well as triggering regulatory functions in gene regulation by their interactions with the different phytohormones. Consequently, we conclude that melatonin, besides being an antioxidant molecule, it is also a key molecule with signaling properties. Beyond scientific interest in the basic research on the complex regulatory function of melatonin and its crosstalk with NO and H_2_O_2_ during the ripening of fruits or their subsequent storage, from an anthropological point of view, one of the stimuli that may promote its use is the nutraceutical benefits fruits enriched in melatonin can provide for human health.
